# A Novel N-Arylpyridone Compound Alleviates the Inflammatory and Fibrotic Reaction of Silicosis by Inhibiting the ASK1-p38 Pathway and Regulating Macrophage Polarization

**DOI:** 10.3389/fphar.2022.848435

**Published:** 2022-03-23

**Authors:** Mingming Fan, Huijuan Xiao, Dingyun Song, Lili Zhu, Jie Zhang, Xinran Zhang, Jing Wang, Huaping Dai, Chen Wang

**Affiliations:** ^1^ Department of Respiratory Medicine, The Second Hospital of Jilin University, Jilin, China; ^2^ Department of Pulmonary and Critical Care Medicine Center of Respiratory Medicine, China-Japan Friendship Hospital, Capital Medical University, National Center for Respiratory Medicine, National Clinical Research Center for Respiratory Diseases, Institute of Respiratory Medicine, Chinese Academy of Medical Sciences, Peking Union Medical College, Beijing, China; ^3^ Department of Pulmonary and Critical Care Medicine, China-Japan Friendship School of Clinical Medicine, Peking University, Beijing, China; ^4^ Institute of Clinical Medical Sciences, China-Japan Friendship Hospital, Beijing, China; ^5^ State Key Laboratory of Medical Molecular Biology, Institute of Basic Medical Sciences Chinese Academy of Medical Sciences, School of Basic Medicine Peking Union Medical College, Beijing, China

**Keywords:** silicosis, pulmonary fibrosis, AKEX0011, macrophage polarization, pirfenidone

## Abstract

Silicosis is one of the potentially fatal occupational diseases characterized by respiratory dysfunction, chronic interstitial inflammation, and fibrosis, for which treatment options are limited. Previous studies showed that a novel N-arylpyridone compound named AKEX0011 exhibited anti-inflammatory and anti-fibrotic effects in bleomycin-induced pulmonary fibrosis; however, it is unknown whether it could also be effective against silicosis. Therefore, we sought to investigate the preventive and therapeutic roles of AKEX0011 in a silicosis rodent model and in a silica-stimulated macrophage cell line. *In vivo*, our results showed that AKEX0011 ameliorated silica-induced imaging lung damages, respiratory dysfunction, reduced the secretion of inflammatory and fibrotic factors (TNF-α, IL-1β, IL-6, TGF-β, IL-4, and IL-10), and the deposition of fibrosis-related proteins (collagen I, fibronectin, and α-SMA), regardless of early or advanced therapy. Specifically, we found that AKEX0011 attenuated silicosis by inhibiting apoptosis, blocking the ASK1-p38 MAPK signaling pathway, and regulating polarization of macrophages. *In vitro*, AKEX0011 inhibited macrophages from secreting inflammatory cytokines and inhibited apoptosis of macrophages in pre-treated and post-treated models, concurrent with blocking the ASK1-p38 pathway and inhibiting M1 polarization. Collectively, AKEX0011, as a novel N-arylpyridone compound, exerted protective effects for silica-induced pulmonary inflammation and fibrosis both *in vivo* and *in vitro*, and hence, it could be a strong drug candidate for the treatment of silicosis.

## Introduction

Silicosis is a form of occupational disease caused by prolonged inhalation of respirable crystalline silica, which is characterized by respiratory dysfunction, chronic interstitial pulmonary inflammation, and diffuse fibrosis (Leung et al., 2012). Although prevention of silicosis can be achieved through appropriate workplace precautions and measurements, the burden of silicosis remains high. Besides the traditional mining industry, there is an emerging epidemic of silicosis within the modern industries such as denim jean production, domestic benchtop fabrication, and jewelry polishing (Barnes et al., 2019; Hoy and Chambers, 2020a; Hoy and Chambers, 2020b). So far, limited progress has been made to elucidate the pathogenesis of silicosis, which restricts its treatment options.

Multiple cell types and complicated molecular signal transduction networks are involved in the cause and progression of silicosis. Silicosis begins with the recruitment of inflammatory cells and excessive secretion of inflammatory and fibrosis factors and chemokines such as tumor necrosis factor-alpha (TNF-α), interleukin-6 (IL-6), interleukin-1 beta (IL-1β), transforming growth factor-beta (TGF-β), matrix metalloproteinases (MMPs), macrophage inflammatory protein, and monocyte chemoattractant protein (Hosseinian et al., 2015; Luna-Gomes et al., 2015; Sayan and Mossman, 2016). The signaling pathways associated with silicosis include phosphatidylinositol 3-kinase ((Chen et al., 2021), RhoA/Rho kinase (Wei et al., 2019), nuclear factor kappa-B (NF-κB) (Di Giuseppe et al., 2009; Abd Elhameed, 2021), mitogen-activated protein kinase (MAPK) (Li et al., 2017), transforming growth factor-beta1/SMAD (Feng et al., 2020), autophagy (Du et al., 2019), and nuclear factor erythroid-related factor2 (Zhu et al., 2020), of which macrophages and their related signaling pathways are widely recognized to play a crucial role in silicosis. Macrophages are gatekeepers of the human body; they can phagocytize particles, activate inflammation, recruit inflammatory cells, and initiate fibrosis (Hosseinian et al., 2015; Luna-Gomes et al., 2015; Sayan and Mossman, 2016). Apoptosis of macrophages is a crucial phenomenon in the pathogenesis of silicosis. Macrophages phagocytose silica but fail to clear them, which leads to cell apoptosis. Apoptotic cells secrete a variety of inflammatory cytokines leading to inflammatory responses (Tan and Chen, 2021a; Tan and Chen, 2021b). The silica particles were further re-released and re-phagocytized, ultimately resulting in the inflammatory cascade reaction (Hu et al., 2006). The persistent inflammatory response eventually leads to the formation of silicosis fibrosis. Macrophages are highly plastic cells that can polarize toward two main subsets: inflammatory macrophages (M1) and anti-inflammatory macrophages (M2) (Xu et al., 2016; Chen et al., 2020). M1 macrophages contribute to the release of pro-inflammatory factors including IL-1β, IL-6, IL-18, IL-23, CCL2, and TNF-α (Tan and Chen, 2021a; Tan and Chen, 2021b); however, M2 subsets can inhibit inflammation and promote tissue repair by secreting anti-inflammatory cytokines such as TGF-β, IL-4, and IL-10 (Tan and Chen, 2021a; Tan and Chen, 2021b). Growing evidence indicates that the macrophage polarization disorder occurs in silicosis because both M1 and M2 macrophage populations increase in experimental silicosis. Therefore, regulating the polarization of macrophages could be an alternative treatment for silicosis (Carneiro et al., 2017; Zhao et al., 2020).

Pirfenidone (PFD) is a pyridine derivative that has been approved by the FDA for the treatment of idiopathic pulmonary fibrosis and has been shown to exert anti-inflammatory and anti-fibrotic effects in multiple fibrosis models. Accumulating *in vivo* and *in vitro* evidence has confirmed that PFD could attenuate the development of silicosis (Cao et al., 2021; Guo et al, 2019). Nevertheless, PFD has significant side effects and other tolerability and half-life (T1/2) issues, which limits its clinical application. Therefore, safe and effective drugs for the treatment of silicosis are urgently needed. AKEX0011 (formerly known as GDC-3280) is an orally available small molecule that was optimized based on PFD’s phenyl pyridone scaffold to improve pharmacokinetics (PK), anti-fibrotic activity, and tolerability over pirfenidone. In preclinical studies, AKEX0011 had better anti-fibrotic activity in lung and liver pharmacological animal models, improved oral bioavailability, and reduced clearance compared to PFD (unpublished data) (Cheung et al., 2021). Therefore, we sought to investigate the role of AKEX0011 in silica-induced pulmonary fibrosis.

Here, we explored the early and advanced therapeutic effectiveness of AKEX0011 in silicosis models using multiple experimental approaches and explored its potential pharmacological mechanisms of action.

## Materials and Methods

### Reagents

AKEX0011 was obtained from Ark Biopharmaceutical Co. Crystalline silica particles were obtained from Sigma-Aldrich (S5631,1-5um, purity 99%). Particulates were baked at 200°C for 3 h and then suspended with phosphate buffer saline (PBS). Silica suspensions were vibrated by sonication for 30 min before use.

### Animal Experiments

Specific pathogen-free (SPF) C57BL/6J (male, 10–11 weeks old, average weight 24–26 g) mice were obtained from Sipeifu Biotechnology (Beijing, China). Mice were fed under SPF conditions with room temperature 20–24°C, humidity 35–55%, and 12/12 h light–dark cycles. These animals were euthanized using intraperitoneal pentobarbital. All animal experiments were approved by the Animal Care and Use Committee of the China-Japan Friendship Hospital (Approval certificate number: zryhyy21-21-01-07).

The silicosis mice model was generated *via* intratracheal instillation of silica using a rodent laryngoscope, which has been previously published (Cao et al., 2020). To establish the early therapeutic model, we randomly divided mice into four experimental groups at 10 animals per group: PBS control, silica + vehicle, silica + AKEX0011, and silica + PFD. PFD was used as the positive control. In the PBS control group, mice were intratracheally instilled with PBS and gavaged with the vehicle (0.5% MC) twice a day. In the silica + vehicle group, mice were instilled with silica and gavaged with the vehicle (0.5% MC) twice a day. In the silica + AKEX0011 group, mice were instilled with silica and gavaged with 100 mpk of AKEX0011 twice a day. In the silica + PFD group, mice were instilled with silica and gavaged with 150 mpk of PFD twice a day. The mice in treatment groups were treated with AKEX0011 or PFD at the day of silica instillation and dosed consecutively for 28 days; then, they were euthanized at day 28.

To perform the advanced therapeutic model, the similar grouping method as early therapeutic models was adapted. We randomly divided mice into four experimental groups at 10 animals per group: PBS control, silica + vehicle, silica + AKEX0011, and silica + PFD. In the PBS control group, mice were intratracheally instilled with PBS and gavaged with the vehicle (0.5% MC) twice a day. In the silica + vehicle group, mice were instilled with silica and gavaged with the vehicle (0.5% MC) twice a day. In the silica + AKEX0011 group, mice were instilled with silica and gavaged with 100 mpk of AKEX0011 twice a day. In the silica + PFD group, mice were instilled with silica and gavaged with 150 mpk of PFD twice a day. Either the vehicle or AKEX0011 or PFD was dosed to mice at day 14 after silica exposure, and then, the drug was dosed consecutively for 28 days; then, they were euthanized at day 42.

### Histological Analysis

The preparation of lung sections for histological analysis was performed, as previously described (Cao et al., 2020). Left lungs were fixed, dehydrated, paraffin-embedded, and then cut into 5 μm sections. Then, the sections were stained by hematoxylin–eosin staining (H&E staining), Masson’s trichrome staining, and Sirius red staining, respectively. H&E staining was undertaken for evaluating the degree of inflammation using Szapiel’s method which consists of no inflammation (grade 0), mild (grade 1+), moderate (grade 2+), and severe (grade 3+) inflammation. The Masson’s trichrome stain was conducted to assess pulmonary fibrosis and was quantified by King’s method (Cao et al., 2020), which is a specific generic method for assessing the silicosis fibrosis scale. Specifically, silicotic nodules on the entire section were defined into five grades (0-5), according to their degree of lesion. Then, the fibrosis score was calculated as the grades (0-5) multiplied by their corresponding percentages of fibrotic area over the total area of the tissue section.

Immunohistochemistry (IHC) analysis was used to detect protein expression levels in lung sections. The lung sections were deparaffinized, rehydrated, and then heated in ethylenediaminetetraacetic acid (EDTA) or citrate buffer. These tissues were blocked by endogenous peroxidase using 0.3% H_2_O_2_ in 5% serum and then incubated with a mixture of anti-collagen I antibody (ab21286, Abcam, United States), anti-fibronectin antibody (ab2413, Abcam, United States), anti-Arginase-1 antibody (Arg1) (ab233548, Abcam, United States), and anti-iNOS antibody (ab178945, Abcam, United States) at 4°C for 12 h and finally added the secondary antibody for 1 h. The positive areas (%) of collagen I, fibronectin, arginase 1, and iNOS staining were measured by Image-Pro Plus 6.0.

### Immunofluorescence Analysis

Frozen sections were blocked by 5% BSA and permeabilized by 0.2% Triton X-100. Then, they were incubated with the anti-alpha smooth muscle actin (α-SMA) antibody (Abcam, ab124964, United States) overnight at 4°C. The fluorescent secondary antibody was added and incubated for 1 h in dark and then mounted with DAPI. The fluorescence microscope (ZEISS, Germany) was used for observation and analysis.

### Lung Function

The flexiVent FX (SCIREQ, Montreal, Quebec, Canada) system was used to assess mouse’s lung functions. On day 28 or 42, after 2% pentobarbital (0.2 ml/100 g) intraperitoneal injection, endotracheal intubations were inserted into tracheas, and a mouse was connected to the ventilator. Parameters of lung functions were obtained, including the inspiratory capacity (IC), respiratory resistance (Rrs), elastic resistance (Ers), and tissue damping (G) (Wu et al., 2020).

### Micro-CT Analysis

Mouse computed tomography scans were performed according to the manufacturer’s instructions with analytic parameters set at 90 kV, 80 mm FOV, 150 mA, and 1.00 mm slice thickness. Cell culture and group.

### Cell Culture Experiments

The mouse macrophage RAW 264.7 cell line was obtained from Procell Biology (CL-0190, Wuhan, China). They were cultured in DMEM (Gibco, 12100046, Carlsbad, United States) with 10% FBS (Gibco, 10091-148, Carlsbad, United States) at 37 C in 5% CO_2_.

As cellular experiments, RAW 264.7 cells were divided into the following: PBS Control, PBS + AKEX0011 (200 μg/ml), silica, silica + AKEX0011 (200 μg/ml), and silica + AKEX0011 (100 μg/ml). As for the pre-treatment experiment, cells in the treatment group were pre-incubated with AKEX0011 for 6 h, and then, silica (100 μg/cm^2^) was added and incubated for 24 h before analysis; cells in the silica group were pre-incubated with PBS for 6 h, and then, silica (100 µg/cm2) was added and incubated for 24 h before analysis. Subsequent mechanism experiments were also conducted in this cell model. As for the post-treatment experiment, cells in the treatment group were incubated with silica (100 μg/cm^2^)for 6 h, and then, AKEX0011 was added and incubated for 24 h before analysis; cells in the silica group were incubated with silica for 30 h.

### ELISA Analysis

The protein expression levels of IL-1β, TNF-α, IL-6, TGF-β, IL-4, and IL-10 in the cell supernatant or BALF and hydroxyproline (HYP) in lung homogenates were measured using ELISA kits. Detailed descriptions are listed in the supplementary materials (Supplementary Table S1).

### Western Blot Analysis

Total proteins of the lung tissue were extracted through RIPA (R0010, Beyotime, Shanghai, China). Their concentrations were determined *via* the BCA Kit (23223, Thermo Scientific, Waltham, United States). For the analysis, 20 µg protein was separated by 8% or 10% SDS-PAGE gels and then electroblotted onto PVDF membranes. The PVDF membranes containing proteins were blocked by 1% BSA for 3 h and then probed using specific primary antibodies at 4°C for 12 h; the antibodies’ information is enumerated in supplementary materials (Supplementary Table S2). Then, we washed membranes using TBST and incubated membranes with secondary antibodies for 1 h. A ChemiDoc XRS + imaging system was used for detection.

### Quantitative PCR Analysis

Total RNAs were extracted from lung tissues and RAW 264.7 *via* TRIzol (15596026, Invitrogen, Carlsbad, United States). Next, we reverse transcribed total RNA into cDNA *via* PrimeScript RT (RR036A, Takara, Shiga, Japan). The qPCR was amplified with a SYBR Green Master Mix Kit (RR420Q Takara, Shiga, Japan) with Bio-Rad IQ5. Related primers are listed in the supplementary information (Supplementary Table S3).

### TUNEL Detection

The apoptotic level in lung tissues was detected by TUNEL staining (C1088, Beyotime, China). Briefly, frozen sections were incubated with 50 μL mixed TUNEL working solution in dark after serum blocking and permeabilization, and then, sections were mounted with DAPI (ab104139, Abcam, United States). A fluorescence microscope (ZEISS, Germany) was used for observation.

### Fluorescence-Activated Cell Sorting Analysis

RAW264.7 cells group as mentioned earlier. The apoptosis detection kit (556547, Becton Dickinson Company, United States) was used following manufacturer’s instructions. Shortly, cells at suitable concentrations were incubated with 5 μl FITC Annexin V and 5 μl PI for 15 min in dark. Then, we analyzed apoptosis by FACS within 1 h. Cells that were stained with FITC Annexin V (+) and PI(+) or (-) were considered apoptotic.

Cells were washed using 3 ml 1% BSA-PBS. Then, we suspended them in 1% BSA-PBS and counted their number with a cell counter, followed by pre-incubation with mouse TruStain FcX PLUS (anti-mouse CD16/32)(Biolegend, 156604, CA, USA)for 10 min. The cell suspension was stained with anti-mouse F4/80 BV605 (743281, Becton Dickinson, San Diego, CA, United States), APC anti-mouse CD163 (155,306, Biolegend, CA, United States), and anti-mouse CD86 BV421(105031, Biolegend, CA, United States) at 4°C for 30 min in the dark. After washing and resuspending in PBS, the cells were analyzed by FACS. The gating strategy was listed in (Supplementary Figure S1).

### Statistical Analysis

Statistics analyses were performed by GraphPad Prism 6.0. Significance of multiple groups used ANOVA, followed by Tukey’s *post hoc* test. A *p*-value less than 0.05 was considered statistically significant. Data were presented as mean ± SEM.

## Results

### AKEX0011 Alleviated Lung Dysfunction, Inflammation, and Fibrosis in the Early Therapeutic Silicosis Model

**FIGURE 1:**
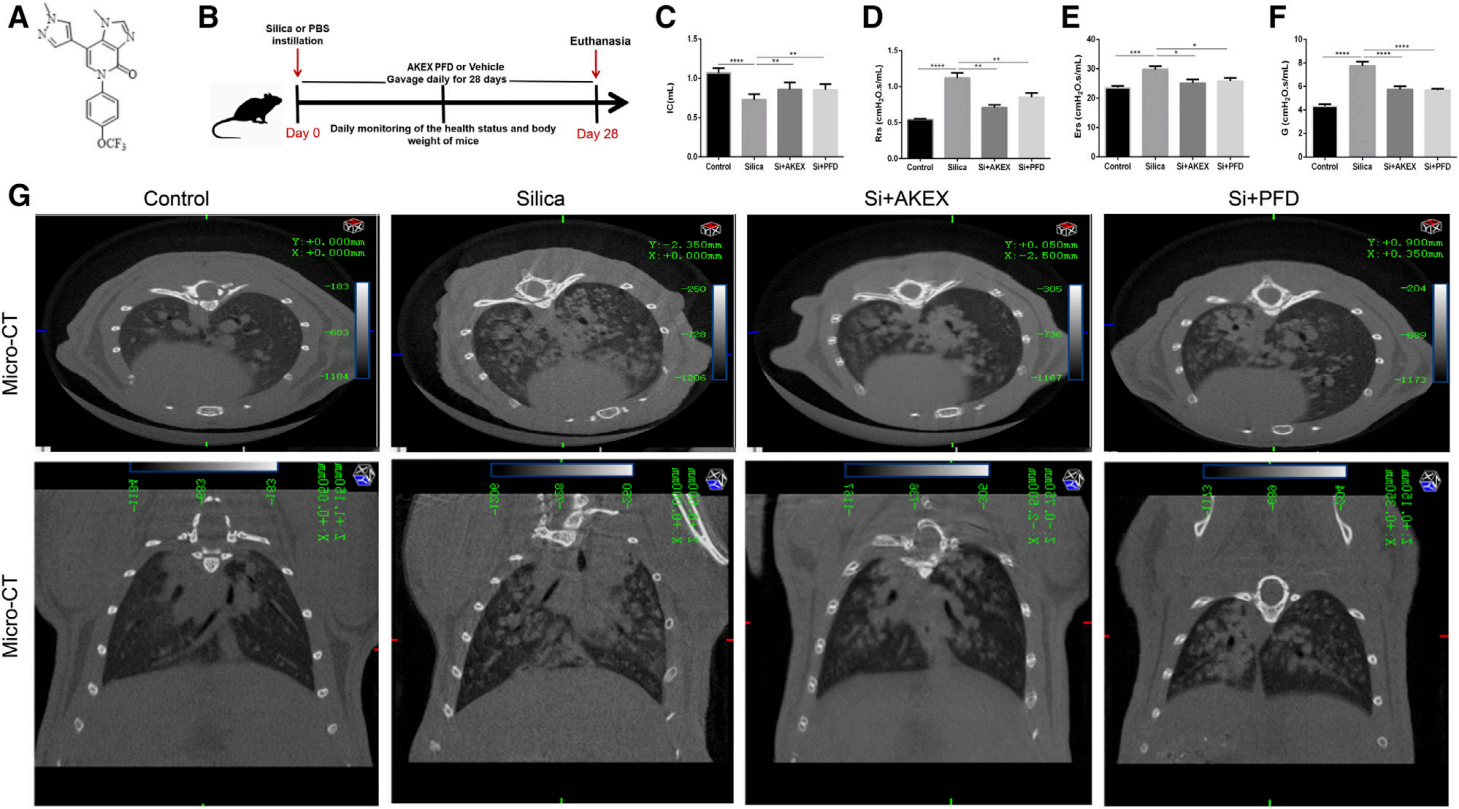
AKEX0011 ameliorated lung dysfunction in the early therapeutic silicosis model. **(A)** Chemical structure of AKEX0011 **(B)** Experimental outline of AKEX0011 treatment in the early therapeutic silicosis model. There were four experimental groups: PBS control (abbreviated as “Control” in the graphs), silica + vehicle (abbreviated as “Silica”), silica + AKEX0011 (abbreviated as “Si + AKEX”), and silica + PFD (abbreviated as “Si + PFD”) in the following experiments of Figures 1 and Figure 4. Silicosis mouse models were established *via* intratracheal instillation of silica suspension (or PBS in control animals). Mice were gavaged with AKEX0011 BID, PFD BID, or vehicle BID after silica instillation and maintained for 28 days; then, they were euthanized at day 28. **(C-F)** Lung function parameters improvement: IC, inspiratory capacity (n = 9-10); Rrs, respiratory resistance (n = 8-9); Ers, elastic resistance (n = 8-10); and G, tissue damping (n = 5-9). **(G)** Chest micro-CT scans. All data were presented as mean ± SEM; *p < 0.05, **p < 0.01, ***p < 0.001, ****p < 0.0001.

To evaluate the protective effect of AKEX0011 (Figure 1A) in the early therapeutic silicosis model (Figure 1B), multiple functional and pathophysiological indicators were assessed including the lung function, micro-CT, and the levels of inflammation and fibrosis. The results from silica-instilled mice were compared to PFD-treated mice, which served as a positive control. We chose the 100 mpk dose of AKEX0011 for the next experiment. AKEX0011 at this dose had a better protective effect and did not alter the lung pathology and lung function, indicating that the 100MPK dose of AKEX0011 had no toxic effect on the lungs (Supplementary Figures S2, S3).

Pulmonary function tests and chest CT are common means of clinical assessment of silicosis. In our study, AKEX0011 treatment alleviated impairment of the lung function and improved lung ventilation and compliance, as indicated by increased inspiratory capacity (IC), decreased respiratory resistance (Rrs), elastic resistance (Ers), and tissue damping (G) (Figures 1C–F). Experimental silicosis mouse micro-chest CT showed round nodules, often symmetrically distributed in the lungs; some nodules fused into a patchy shadow. These damages reduced the lung volume and transmittance and imaging damages that have been prominently alleviated with AKEX0011 therapy (Figure 1G).

**FIGURE 2:**
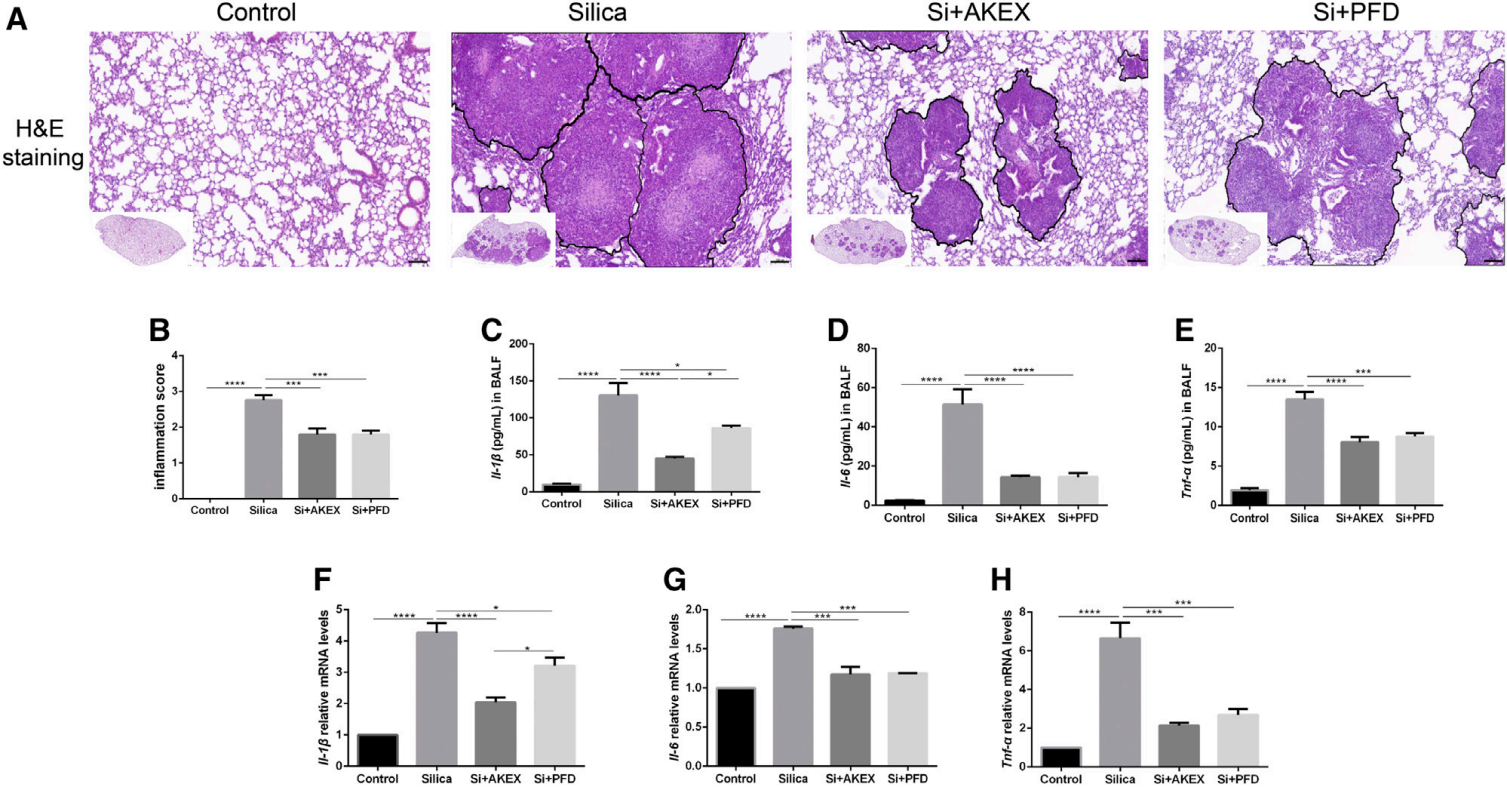
AKEX0011 reduced inflammation in the early therapeutic silicosis model. **(A)** H&E staining of lung sections (original magnification ×100). **(B)** Inflammatory score (based on H&E staining) (n = 5). **(C-E)** Inflammatory cytokines in the BALF (IL-1β, IL-6, and TNF-α) measured by ELISA (n = 5). **(F-H)** Levels of inflammatory cytokines (IL-1β, IL-6, and TNF-α) in lung tissues measured by qPCR (n = 3-4). All data were presented as mean ± SEM; *p < 0.05, **p < 0.01, ***p < 0.001, and ****p < 0.0001.

Persistent inflammation is an important characteristic of silicosis. We evaluated effects of AKEX0011 in the inflammatory reaction of silicosis. H&E staining showed that compared to the PBS control group, the lungs of silica + vehicle mice exhibited nodules in different sizes, alveolar collapse, thickened alveolar septa, inflammatory cell infiltration, and interstitial edema. These pathological changes were alleviated after AKEX0011 treatment (Figures 2A,B). Moreover, AKEX0011 treatment reduced the protein expression of pro-inflammatory factors including TNF-α, IL-6, and IL-1β in the bronchoalveolar lavage fluid (BALF), compared with the silica + vehicle group (Figures 2C–E). These findings were consistent with their mRNA expressions in lung tissues (Figures 2F–H). Taken together, our results indicate that the early therapy of AKEX0011 attenuated an inflammatory response in the silicosis mouse model.

**FIGURE 3:**
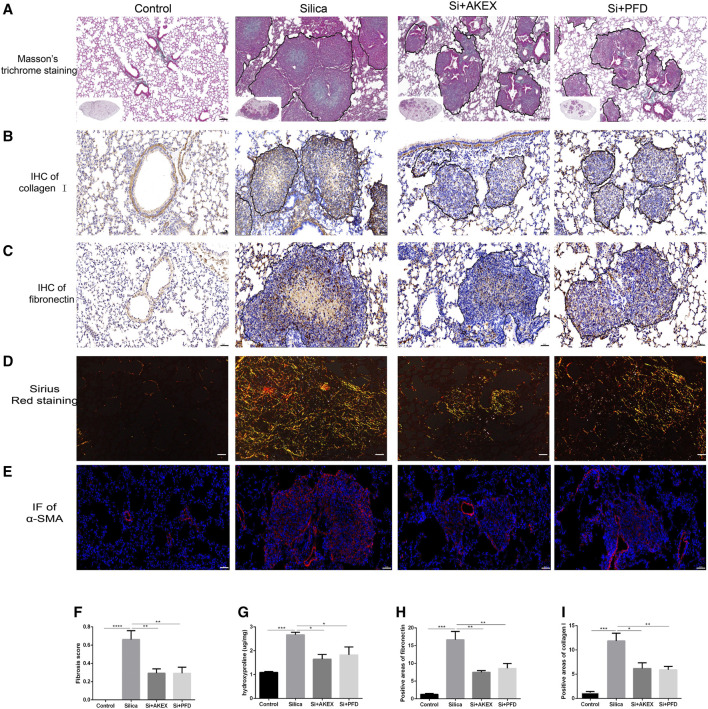
AKEX0011 ameliorated pulmonary fibrosis in the early therapeutic silicosis model. **(A)** Masson’s trichrome staining of lung sections (original magnification ×100). (**B,C)** IHC staining of collagen I and fibronectin on lung sections (original magnification ×200). **(D)** Sirius red staining on lung sections (original magnification ×200). **(E)** IF staining of α-SMA on lung sections (original magnification ×200). **(F)** Fibrosis score based on Masson’s trichrome staining (n = 5). **(G)** Levels of HYP in lung tissues (n = 3 or 5). **(H,I)** Quantification of collagen I and fibronectin-positive areas based on images in (B,C) (n = 4). All data were presented as mean ± SEM; *p < 0.05, **p < 0.01, ***p < 0.001, and ****p < 0.0001.

**FIGURE 4:**
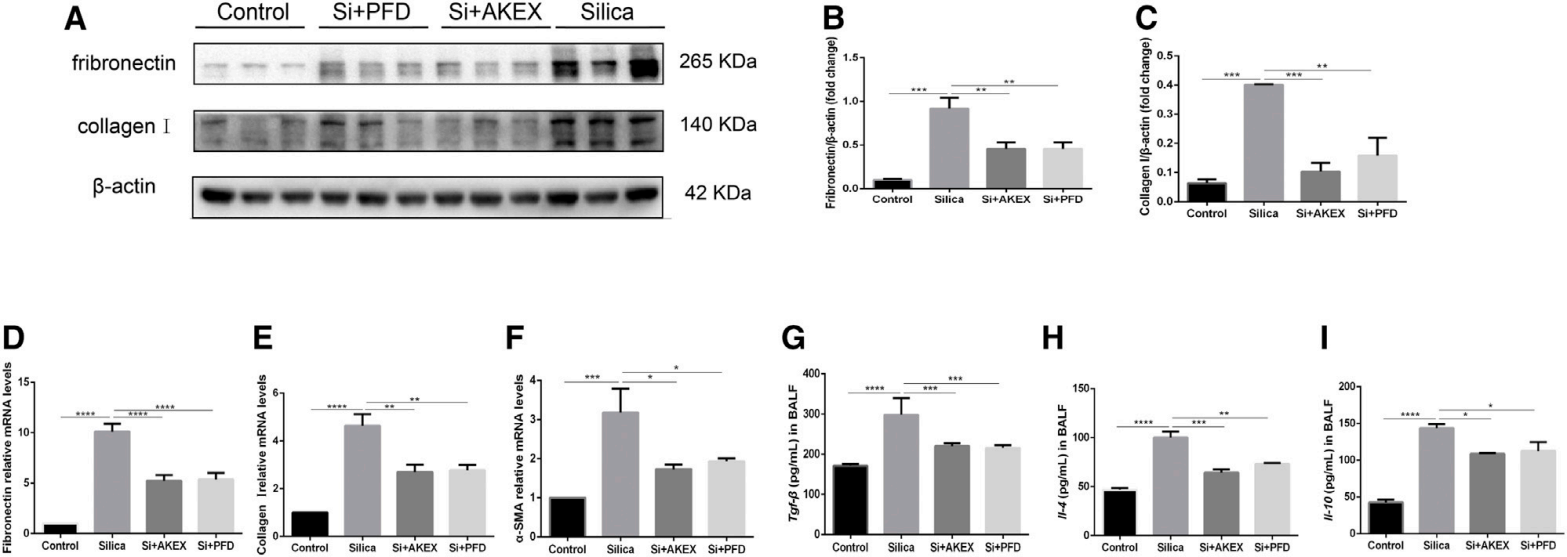
AKEX0011 ameliorated pulmonary fibrosis in the early therapeutic silicosis model. **(A)** WB of fibronectin and collagen I. β-actin was used as a loading control. **(B,C)** Quantification of band densities from images in (A). **(D-F)** mRNA levels of fibronectin, collagen I, and α-SMA in lung tissues were detected by qPCR(n = 5). **(G-I)** TGF-β, IL-4, and IL-10 in the BALF detected by ELISA (n = 3-5). All data were presented as mean ± SEM; *p < 0.05, **p < 0.01, ***p < 0.001, and ****p < 0.0001.

We next evaluated the extent of pulmonary fibrosis between the untreated and AKEX0011-treated groups using several methods. Masson’s trichrome and Sirius red staining demonstrated less extracellular matrix (ECM) deposition in silica + AKEX0011 mice than in silica + vehicle mice (Figures 3A,D,F). Accordingly, the amount of HYP in the tissue homogenate was downregulated upon AKEX0011 treatment (Figure 3G). Furthermore, decreased deposition levels of collagen I, fibronectin, and α-SMA in IHC or IF staining indicated the reduction of fibrosis (Figures 3A–I). Consistent with these results, protein and/or mRNA levels of collagen I and fibronectin additionally confirmed the attenuation of fibrosis in the AKEX0011 treatment group in comparison to the untreated group (Figures 4A–F). It is known that TGF-β is tightly linked to fibroblast activation and fibrogenesis, whereas TGF-β is tightly linked to fibroblast activation and fibrogenesis (Du et al., 2019); meanwhile, IL-4 and IL-10 are important pro-fibrotic factors. ELISA results demonstrated a reduced protein expression of TGF-β, IL-4, and IL-10 in the BALF in AKEX0011-treated mice compared to the untreated ones (Figures 4G–I).

To summarize, these findings revealed that AKEX0011 improved lung dysfunction, decreased inflammation, and attenuated fibrosis in the early therapeutic silicosis model. Noticeably, AKEX0011 treatment had a stronger effect in reducing the IL-1β level compared to PFD treatment. For other inflammatory and fibrotic biomarkers, AKEX0011 treatment tends to have a comparative effect to the positive control of PFD.

### AKEX0011 Ameliorated Pulmonary Inflammation and Fibrosis in the Advanced Therapeutic Silicosis Model

**FIGURE 5:**
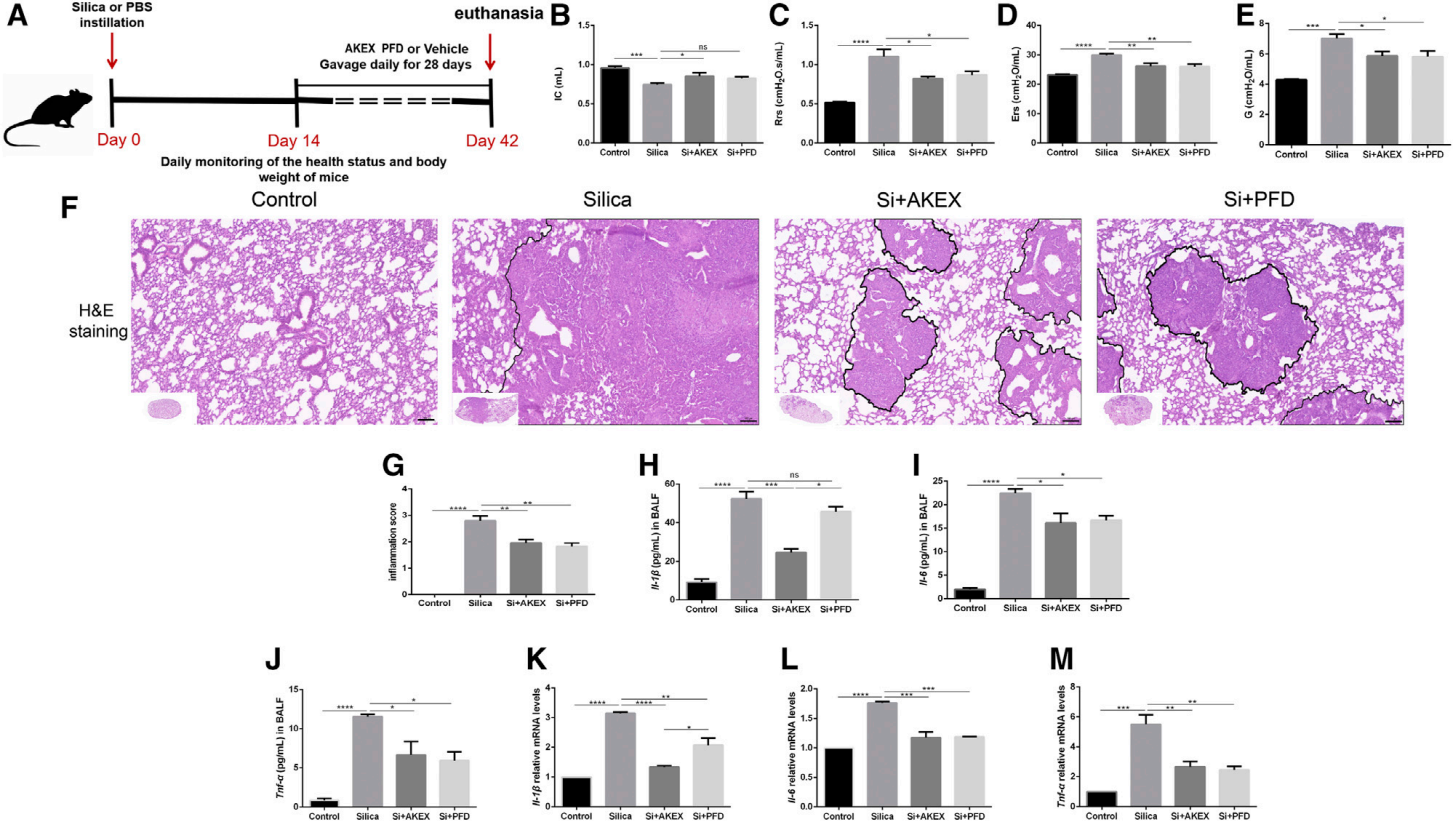
AKEX0011 ameliorated lung function and reduced pulmonary inflammation in the advanced therapeutic silicosis model. **(A)** Experimental outline was depicted. Silicosis models were established as mentioned earlier. Mice were gavaged with AKEX0011, PFD, or vehicle at day 14 after silica exposure and drug administration for 28 days; then, they were euthanized at day 42. There were four experimental groups: PBS control (abbreviated as “Control” in the graphs), silica + vehicle (abbreviated as “Silica”), silica + AKEX0011 (abbreviated as “Si + AKEX”), and silica + PFD (abbreviated as “Si + PFD”) in Figure 5 and Figure 6. **(B-E)** Lung function test; IC, inspiratory capacity (n = 7-9); Rrs, respiratory resistance (n = 5-8); Ers, elastic resistance (n = 5-8); and G, tissue damping (n = 5-7). **(F)** H&E staining on lung sections (original magnification ×100). **(G)** Inflammatory score (based on H&E staining) (n = 5). **(H-J)** IL-1β, IL-6, and TNF-α in the BALF detected by ELISA (n = 3). **(K-M)** mRNA levels of IL-1β, IL-6, and TNF-α in lung tissues were detected by qPCR(n = 3). All data were presented as mean ± SEM; *p < 0.05, **p < 0.01, ***p < 0.001, and ****p < 0.0001.

**FIGURE 6:**
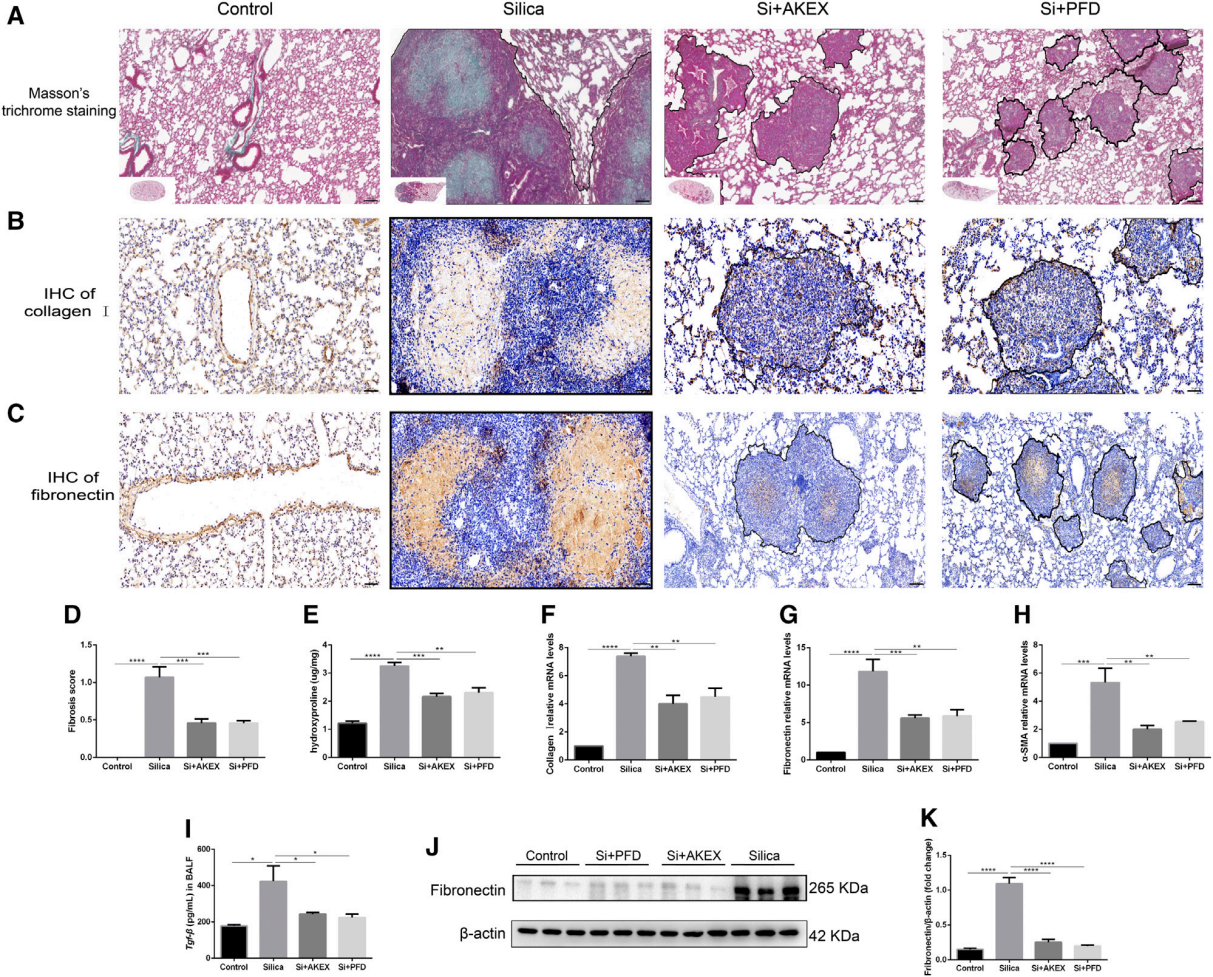
AKEX0011 reduced pulmonary inflammation and fibrosis in the advanced therapeutic silicosis model. **(A)** Masson’s trichrome staining on lung sections (original magnification ×100). **(B,C)** IHC staining of collagen I and fibronectin on lung sections (original magnification ×100) (n = 3). **(D)** fibrosis score (based on Masson’s trichrome staining) **(E)** levels of HYP in lung tissues (n = 3). **(F-H)** mRNA levels of collagen I, fibronectin, and α-SMA in lung tissues detected by qPCR(n = 3). **(I)** TGF-β in the BALF detected by ELISA (n = 3). **(J)** WB of fibronectin. β-actin was used as a loading control. **(K)** Quantification of band densities from images in (j). All data were presented as mean ± SEM; *p < 0.05, **p < 0.01, ***p < 0.001, and ****p < 0.0001.

In clinical practice, early diagnosis of silicosis is very challenging; hence, silicosis patients are often diagnosed long after the silica exposure. Therefore, we further investigated whether advanced therapy of AKEX0011 could ameliorate experimental silicosis. So we gavaged mice with AKEX0011 or PFD at day 14 after silica exposure and kept drug dosing for the subsequent 28 days. The same strategy, as mentioned earlier, was used to evaluate the long-term efficacy of AKEX0011 (Figure 5A). In this study, AKEX0011 significantly alleviated the impairment of the lung function, as indicated by the amelioration of IC, Rrs, Ers, and G parameters (Figures 5B–E). Similarly, H&E staining revealed that inflammatory infiltration was significantly reduced upon AKEX0011 treatment compared to the silica vehicle-only mice (Figures 5F,G). The silica-induced increase of the levels of inflammatory factors (TNF-α, IL-6, and IL-1β) in the BALF and lung tissues was remarkably reduced in the AKEX0011 treatment group (Figure 5H-5M). Furthermore, Masson’s trichrome and IHC staining and HYP measurements demonstrated that ECM deposition was decreased after AKEX0011 treatment (Figures 6A–E), indicating attenuated pulmonary fibrosis. In line with this observation, attenuated silicosis was confirmed by the decreased expression of fibronectin, collagen I, α-SMA, and TGF-β (Figures 6F–K). In conclusion, our results indicate that AKEX0011 improved the lung function, alleviated pulmonary inflammation, and delayed fibrosis even in the advanced therapeutic silicosis model.

### AKEX0011 Ameliorated Silicosis Models by Blocking the ASK1-p38 MAPK Signaling Pathways and Inhibiting Apoptosis

**FIGURE 7:**
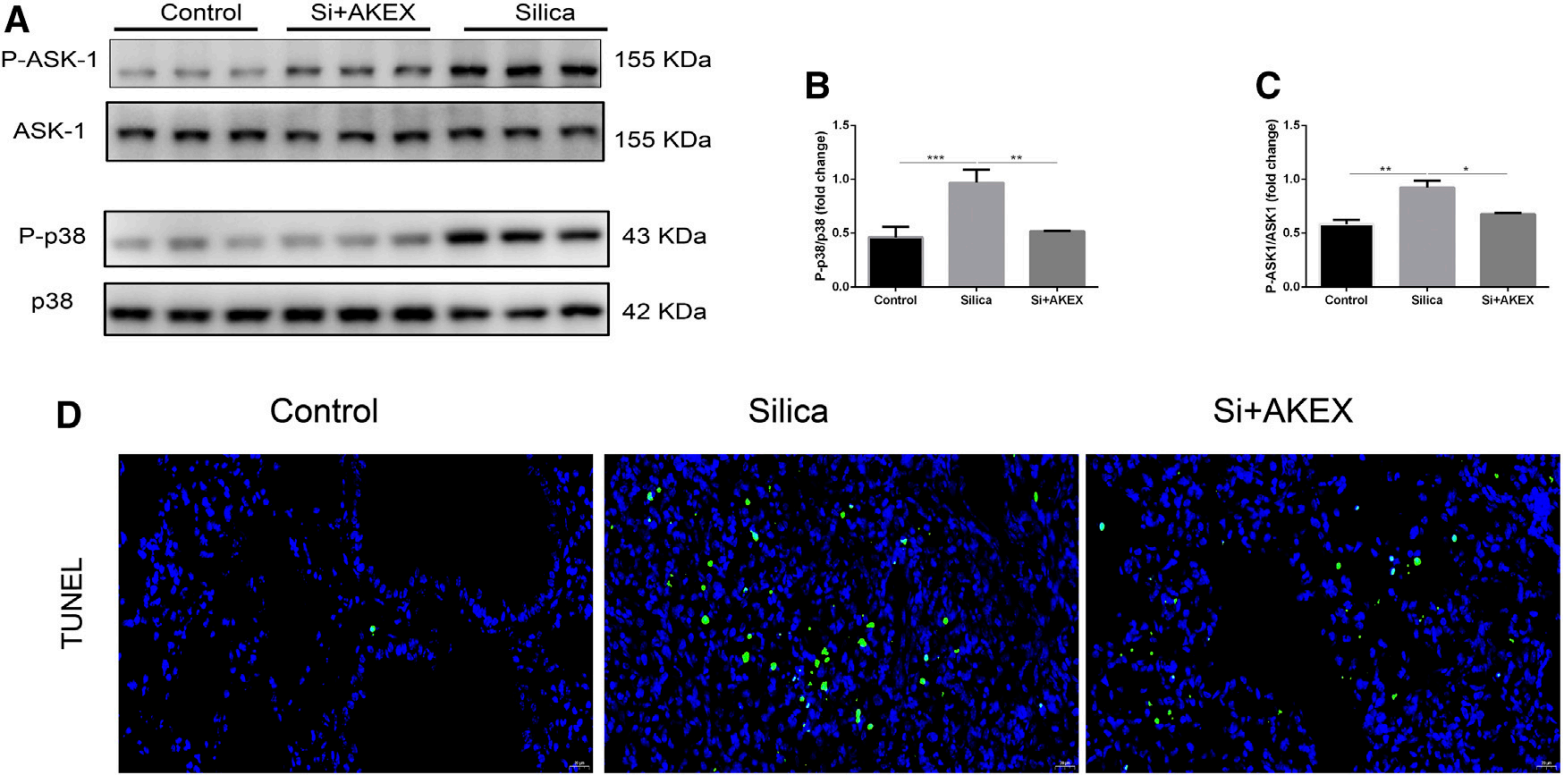
AKEX0011 inhibited inflammation and fibrosis by blocking the ASK-p38 MAPK signaling pathways and apoptosis. There were three experimental groups: the PBS control group (abbreviated as “Control” in the graphs), silica + vehicle group (abbreviated as “Silica”), and silica + AKEX0011 group (abbreviated as “Si + AKEX”) in the experiments of Figure 7 and Figure 8. **(A)** WB of P-ASK1, ASK1, P-p38, and p38. **(B-C)** Quantification of band densities from WB images in (A). **(D)** TUNEL staining. All data were presented as mean ± SEM; *p < 0.05, **p < 0.01, and ***p < 0.001.

After confirmation of the therapeutic effect of AKEX0011 in treatment of silicosis, we further investigated its mechanisms of action. MAPKs perform a vital regulatory role in the development of inflammation and fibrosis. p38, a subclass of MAPKs, is regulated by apoptosis signal-regulating kinase 1 (ASK1), which is an upstream activator of p38. ASK1/p38 is involved in several fibrotic diseases including silicosis (Du et al., 2019). We studied AKEX0011 effects on the ASK1-p38 MAPK signal transduction pathway. Our studies showed that WB results demonstrated that exposure to silica significantly increased the phosphorylation of ASK1 and p38 in mice, whereas AKEX0011 treatment inhibited these changes (Figures 7A–C). These findings suggest that AKEX0011 might exert anti-inflammatory and anti-fibrotic effects by blocking the ASK1-p38 MAPK pathway.

Since apoptosis closely linked to silicosis, we performed TUNEL staining to detect apoptosis in the lung sections. We observed that the number of TUNEL-positive cells increased in silica + vehicle mice, while it decreased in silica + AKEX0011 mice (Figures 7D,E).

### AKEX0011 Regulated Polarization of Pulmonary Macrophages in Silicosis Mice

**FIGURE 8:**
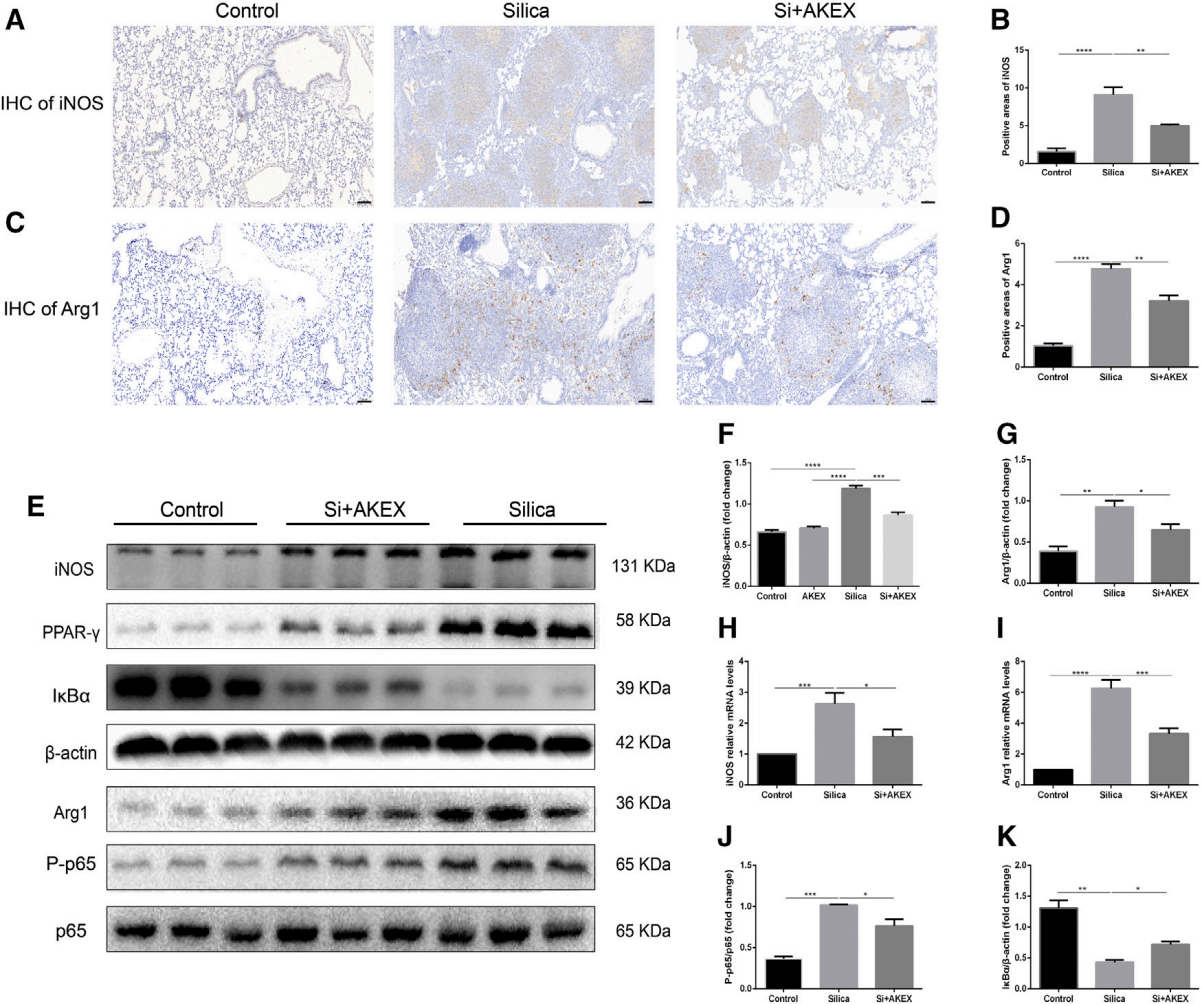
AKEX0011 regulated polarization of pulmonary macrophages in the silicosis mouse model. **(A,B)** IHC staining and quantification of the M1 macrophage marker iNOS (original magnification ×100) (n = 3). **(C,D)** IHC staining and quantification of the M2 macrophage marker Arg1 (original magnification ×100) (n = 3). **(E)** WB of iNOS, p65, P-p65, PPAR-γ, IκBα, and Arg1. β-actin was used as a loading control. **(F,G,J,K)** Quantification of band densities from WB images in (e). **(H,I)** mRNA levels of iNOS and Arg1 in lung tissues detected by qPCR(n = 4-5). All data were presented as mean ± SEM; *p < 0.05, **p < 0.01, and ***p < 0.001.

Macrophages play a key role in the development of silicosis as many previous studies demonstrated that activation of macrophages and their polarization into M1 and M2 subtypes promoted silicosis development (Carneiro et al., 2017; Zhao et al., 2020; Liu et al., 2021). To verify if this was also the case in the silicosis mouse model, we stained iNOS and Arg1, specific markers of M1 and M2, respectively. IHC on lung sections confirmed increased activation of both M1 and M2 macrophages in the silica group; the increase in the M1 subtype was more pronounced; administration of AKEX0011 reduced the accumulation of M1 and M2 macrophages, as compared to the silica group (Figures 8A–D). Furthermore, mRNA and protein levels of iNOS and Arg1 were significantly increased in the silica group, in comparison to the PBS control group, and AKEX0011 decreased the expression of these markers (Figures 8E–I). Additionally, the expressions of M1-related inflammatory factors (IL-1β, IL-6, and TNF-α) and M2-related fibrotic factors (IL-4, TGF-β, and IL-10) were also reduced upon AKEX0011 treatment.

Previous studies demonstrated that the NF-κB (p65)/IκBα pathway, of which IκBα is the major NF-κB inhibitor protein, controls M1 macrophage polarization ((Zhao et al., 2020; Carneiro et al., 2017; Liu et al., 2021)). Hence, we explored whether AKEX0011 could regulate this pathway. WB analysis revealed that silica significantly increased the ratio of the phosphorylated NF-κB to the total NF-κB level (P-NF-κB/NF-κB) and reduced the expression level of IκBα. AKEX0011 treatment reduced the P-NF-κB/NF-κB ratio and elevated the expression of IκBα, indicating that AKEX0011 might inhibit M1 macrophage polarization through blocking of NF-κB signaling (Figures 8E,J,K). Also, PPAR-γ is a vital molecule controlling M2 macrophage polarization ((Carneiro et al., 2017; Zhao et al., 2020; Liu et al., 2021)). We demonstrated that PPAR-γ was significantly upregulated in the silica group, and AKEX0011 reversed this upregulation (Figure 8E), thus surmising that AKEX0011 attenuates M2 macrophage polarization through PPAR-γ inhibition. PPAR-γ and NF-κB can affect each other. Usually, PPAR-γ activation causes NF-kB inhibition. We believe that AKEX affects these two pathways independently, resulting in an interaction between the two pathways which is not obvious.

### AKEX0011 Inhibited Secretion of Cytokines From Macrophages and Apoptosis in Pre-Treated and Post-Treated Cell Models

To further explore AKEX0011 mechanisms of action at the cellular level, we performed *in vitro* experiments. In view of the important role of macrophages in the pathogenesis of silicosis and the protective effect of AKEX0011 on macrophage *in vivo* experiments*,* we used RAW264.7 macrophages for *in vitro* experiments. We used silica-stimulated macrophages and treated them with different concentrations of AKEX0011 at different timings, as described in the materials and methods.

**FIGURE 9:**
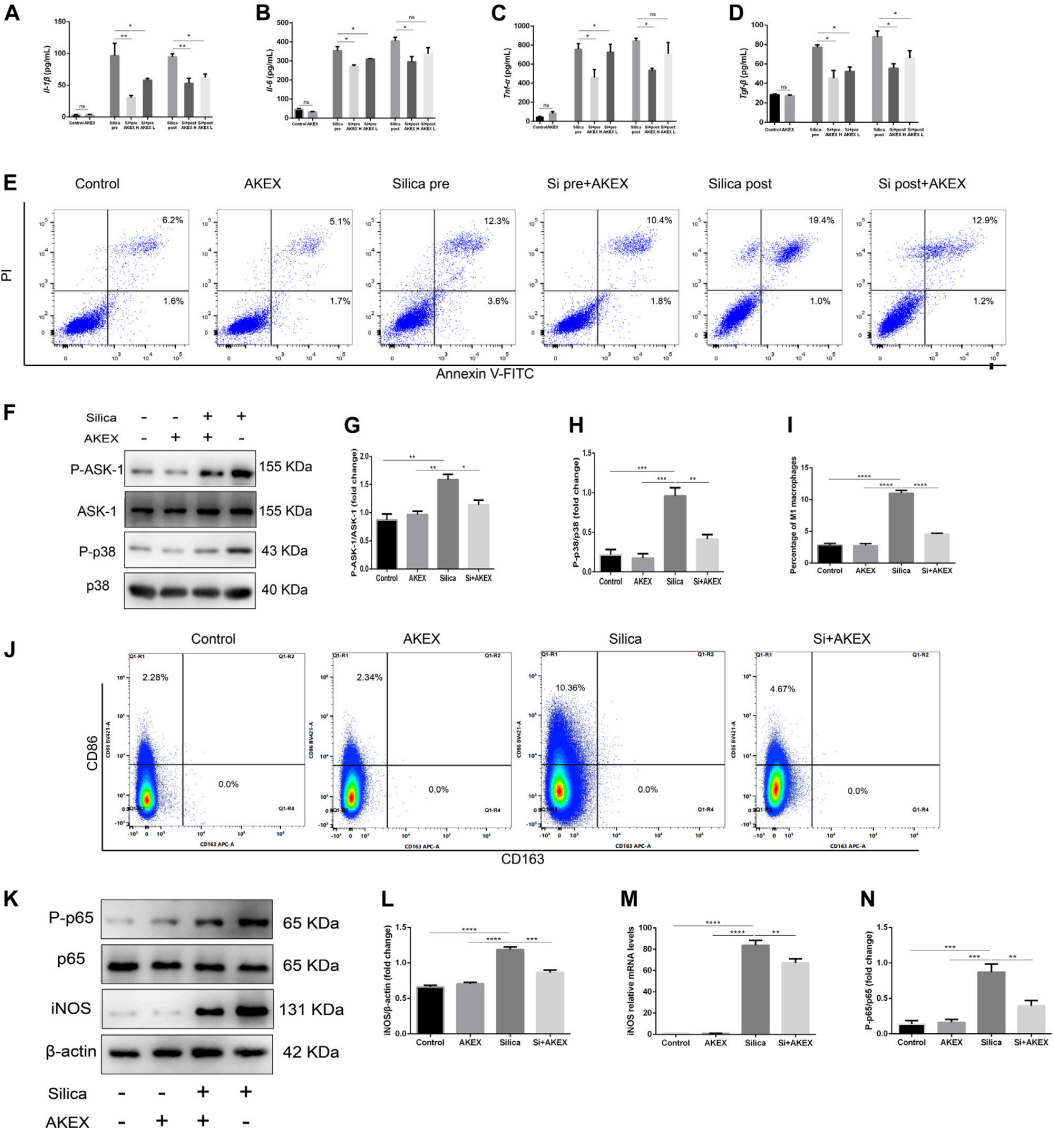
AKEX0011 inhibited RAW264.7 from secreting pro-inflammatory cytokines, blocked p38 MAPK signaling, and reduced silica-induced apoptosis and M1 polarization. There were eight cell groups: PBS Control (abbreviated as “Control” in the graphs), PBS + AKEX0011 (200 μg/ml) (abbreviated as “AKEX”), Silica pre, Silica pre + AKEX0011 (100 μg/ml) (abbreviated as “Si pre + AKEX L”), and Silica pre + AKEX0011 (200 μg/ml) (abbreviated as “Si pre + AKEX H”), Silica post, Silica post + AKEX0011 (100 μg/ml) (abbreviated as “Si post + AKEX L“), and Silica post + AKEX0011 (200 μg/ml) (abbreviated as “Si post + AKEX H”) **(A–D)** IL-6 IL-1β, TNF-α, and TGF-β in cell supernatant detected by ELISA (n = 3). **(E)** Apoptosis (Annexin V+/PI– and Annexin V+/PI+) detection by FACS in each experimental group. **(F–H)** WB and quantification of P-p38, p38, P-ASK1, and ASK1. β-actin was used as a loading control. **(I–J)** M1 (F4/80 + CD86+) and M2 (F4/80 + CD163+) macrophage proportions detected by FACS in RAW264.7 and statistical analysis (n = 3). **(K)** WB of iNOS P-p65 p65. **(L** and **N)** Quantification of band densities from WB images in (k), (n = 3). **(M)** mRNA levels of iNOS in lung tissues detected by qPCR (n = 3). All data were presented as mean ± SEM; *p < .05, **p < .01, ***p < .001, and ****p < .0001.

To study preventive and therapeutic effects of AKEX0011 on the secretion of cytokines in the macrophages, we measured the levels of major cytokines in the cellular supernatant by ELISA. Our results demonstrated that AKEX0011 effectively inhibited silica-induced macrophages from the secretion of IL-1β, IL-6, TNF-α, and TGF-β; the therapeutic effects of AKEX0011 are better in the high-dose group, so we chose high-dose AKEX0011 treatment for the next experiment (Figures 9A–D). We investigated whether AKEX0011 could inhibit macrophages from silica-induced apoptosis. FACS analysis showed that silica can induce macrophage apoptosis, especially late apoptosis. In the pre-treated experiment, it can be seen that a small proportion of macrophages undergo early apoptosis, while in the post-treated experiment, almost all of macrophages undergo late apoptosis. Pre- and post-AKEX0011 treatment specifically decreased silica-induced cell apoptosis (Figure 9E), suggesting that AKEX0011 can ameliorate silicosis by inhibiting apoptosis.

### AKEX0011 Blocked ASK1-p38 MAPK Signaling and Reduced Silica-Induced M1 Macrophage Polarization

Previous studies have shown that silica inducing secretion of cytokines in macrophages is partly through ASK1-p38 MAPK pathways (Carneiro et al., 2017; Zhao et al., 2020; Liu et al., 2021). In our study, WB analysis revealed that AKEX0011 effectively blocked the activation of the ASK1-p38 MAPK signaling pathway in macrophages exposed to silica (Figures 9F–H). So we speculate that AKEX0011 ameliorates silicosis by inhibiting ASK1-p38 MAPK pathways in macrophages.

Since silica can induce polarization of RAW264.7 macrophages toward certain M1 subtypes, FACS analysis revealed that the ratio of F480 + CD86 ^+^ M1 macrophages significantly increased after silica exposure for 24 h, while the ratio of F480 + CD163 + M2 macrophages did not change (Figures 9I–J) compared to the PBS control group. Consistently, mRNA and protein expressions of iNOS increased after silica exposure. We treated macrophage cells with AKEX0011 and found that it decreased the ratio of F480 + CD86 ^+^ M1 macrophages (Figures 9I–J) and the expression of iNOS, as compared to the silica group (Figures 9K–M). WB analysis revealed that AKEX0011 effectively blocked the activation of the NF-κB signaling pathway in macrophages exposed to silica (Figure 9N). Meanwhile, we have shown that the level of M1-related inflammatory factors IL-1β, IL-6, and TNF-α was reduced by AKEX0011 treatment as well. Altogether, our findings indicated that AKEX0011 can inhibit M1 polarization *via* the NF-κB signaling pathway in silica-induced macrophages.

## Discussion

Silicosis mouse models in the current study were generated *via* intratracheal instillation of silica. We demonstrated that the lung function pathologies and histological abnormalities in our model resembled those occurring in human silicosis. Histopathology of silicosis mouse models exhibited silicotic nodules with central fibrosis and peripheral infiltration of inflammatory cells. Lung function tests showed a restrictive ventilatory disorder and aberrant lung resistance and compliance (Cao et al., 2020). AKEX0011 significantly alleviated histopathological defects and impairment of lung functions in early and advanced therapeutic silicosis mouse models. AKEX0011 also significantly decreased the production of inflammatory factors such as TNF-α, IL-1β, IL-6, and TGF-β and the production of fibrosis markers such as collagen I, fibronectin, and α-SMA in silicosis.

Inflammation is an integral part of the pathogenesis of silicosis. Macrophages are an important cause of this uncontrolled inflammation. Macrophages recognize and phagocytose silica, leading to a series of inflammatory reactions such as activation of inflammatory factors, inflammasomes, and other inflammation-related pathways. In our study, AKEX0011 showed a significant inhibitory effect on silica-induced inflammation reactions both *in vivo* and in RAW264.7 macrophages. Specifically, the inhibitory effect of AKEX0011 on IL-1β secretion was superior to the one on the PFD-positive control. IL-1β is a cytokine derived from the mononuclear phagocyte system and plays an important role during silicosis. It has been demonstrated that IL-1β secretion aggravated silicosis, neutralization, and blocking of IL-1β, which could reverse silicosis (Hoffman and Wanderer, 2010; Guo et al., 2013). In addition, the importance of IL-1β has also been implicated in clinical patients, and IL-1 gene polymorphism may confer an increased risk for the development of silicosis (Rao et al., 2014; Luna-Gomes et al., 2015). There was a clinical case report which showed that the IL-1 receptor blockade alleviated silicosis (Frank and Vince, 2019). Moreover, besides its direct effect, IL-1β can indirectly affect silicosis by acting on its downstream mediators. CXC chemokines are products of IL-1β that attract neutrophils to the epithelium, where they can cause further damage and initiate fibrosis (Liu, 2008). Furthermore, IL-1β can enhance the production of TGF-β, the core protein of fibrotic diseases closely linked to fibrogenesis. TGF-β can activate epithelial cell and fibroblast proliferation and conversion into myofibroblasts (dos Santos et al., 2012). Hence, the reduction of the TGF-β level by AKEX0011 treatment may be partly due to the reduction of the IL-1β level. Polarization of macrophages plays key roles in the pathogenesis of pulmonary fibrosis. M1 macrophages secrete IL-1β, IL-6, and TNF-α, which may lead to lung inflammatory diseases, whereas M2 macrophages produce TGF-β, IL-4, and IL-10, inhibiting the M1 response and are associated with fibrogenesis (Wang et al., 2014; Wynn and Vannella, 2016). Previous studies and present research demonstrated that the polarization into M1/M2 subtypes and M1/M2-related inflammatory factor levels are increased in silicosis. These specific changes are closely related to the particular silicosis microenvironment. The microenvironment of lung tissue in silicosis is unique because of continuous stimulation by silica particles and the co-existence of progressive inflammation with fibrosis. Macrophage polarization may be one of the causes of the formation of this microenvironment; however, the changes in the microenvironment itself may also influence the polarization of macrophages. There are studies investigating the dynamic changes of these processes and their microenvironment in silicosis (Wang et al., 2014; Wynn and Vannella, 2016). Specifically, after the silica exposure, the proportion of M1 macrophages detected by FACS promptly increased and remained at a high level. However, the increase in M1-related inflammatory cytokine (IL-1β, IL-6, and TNF-α) levels lagged behind (Wang et al., 2014; Wynn and Vannella, 2016), suggesting that silica triggered macrophage polarization and initiated the inflammatory microenvironment. When the damage caused by silica accumulated to a certain extent, wound healing was initiated; macrophages gather at the injured pulmonary tissue and participate in tissue repair. Fibrogenic factors IL-4, IL-10, and TGF-β increased, subsequently, the proportion of M2 macrophages. A large number of M2 macrophages together with prolonged inflammation and increased levels of pro-fibrotic factors maintain the fibrosis microenvironment and exacerbate silicosis fibrosis. AKEX0011 inhibited M1 polarization and in the meantime limited M2 polarization, reduced secretion of M1-and M2-related cytokines, alleviated microenvironmental disorders, and subsequently ameliorated pulmonary fibrosis. However, in the *in vitro* studies, silica stimulation induced M1 polarization instead of M2 polarization, which could be due to the lack of the M2 polarization microenvironment; thus, a more accurate cell model is needed to better recapitulate the *in vivo* environment. Although there is no M2 polarization under silica stimulation *in vitro*, various *in vitro* experiments have shown that silica can facilitate the secretion of pro-fibrotic factors in macrophages to aggravate pulmonary fibrosis (Pang et al., 2021). Silica-exposed macrophage-derived exosomes can activate fibroblasts to proliferate and to express collagen I and α-SMA (Wang et al., 2020; Qin et al., 2021). Our experiments also proved that silica can induce macrophages secreting TGF-β, and AKEX0011 can alleviate this effect. The p38 MAPK pathway is pivotal in the development of silicosis; the p38 kinase inhibitor (SB203580) can ameliorate pulmonary inflammation and fibrosis in the rat silicosis model (Wu et al., 2021). Meanwhile, studies have shown that inhibition of the ASK1-p38 pathway can significantly inhibit the secretion of inflammatory factors in silica-induced macrophages (Wang et al., 2020; Qin et al., 2021). AKEX0011 can inhibit the ASK1-p38 pathway both *in vivo* and *in vitro*, so we concluded AKEX0011 might exert anti-inflammatory and anti-fibrotic effects by blocking the ASK1-p38 MAPK pathway. These potential mechanisms influence and regulate each other and together constitute the biological effect of AKEX0011.

PFD has proven its anti-inflammatory, antioxidant, and anti-fibrotic effects in many fibrotic diseases. Since PFD reduced the severity of silicosis by inhibiting the epithelial mesenchymal transition (EMT), regulating the TGF-β 1/Smad2/3 signaling pathway (Wang et al., 2020; Qin et al., 2021), and inhibiting the production of IL-17A (24), we used it as a positive control in our study. AKEX0011 is not inferior to PFD in the prevention and treatment of silicosis. Besides, PFD has significant side effects in patients, and the most commonly reported adverse events include GI- and skin-related. The effective treatment is compromised due to these adverse effects and management of them through optimal dose-response adjustments (Lancaster et al., 2016). Therefore, more efficacious medications are needed to treat fibrosis. AKEX0011 outperformed PFD in drug toxicology (unpublished data). A phase 1, randomized study in healthy subjects demonstrated an AKEX0011-ranged oral dose of 25–1,600 mg has a favorable safety and pharmacokinetic profile. Overall, AKEX0011 could be a promising candidate for the treatment of silicosis-associated pulmonary fibrosis.

## Conclusion

**FIGURE 10:**
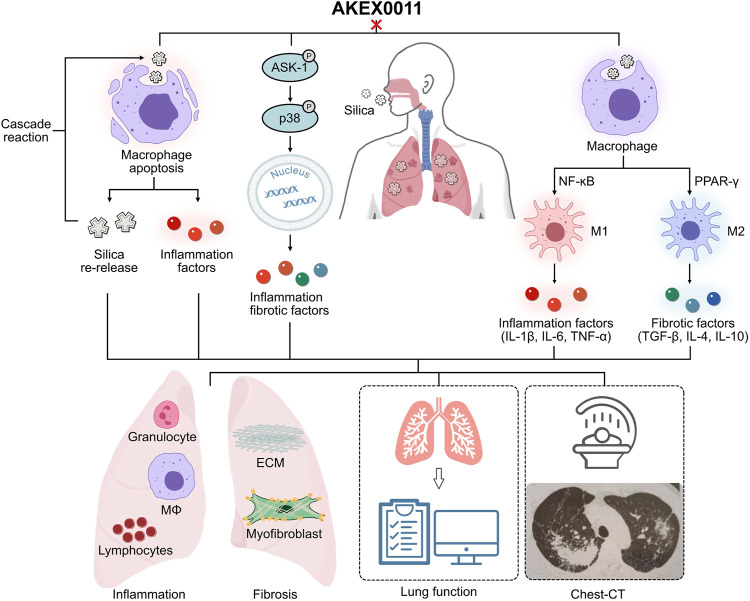
Ideograph of AKEX0011’s therapeutic effects. AKEX0011 alleviated the inflammatory and fibrotic reactions of silicosis through the following mechanisms **(A)** Apoptosis: AKEX0011 reduced silica-induced apoptosis and alleviated the inflammatory cascade caused by apoptosis, which ultimately reduced the extent of silicosis. **(B)** ASK1-p38 MAPK signaling pathway: silica can induce inflammation and fibrosis by activation of the ASK1-p38 MAPK signaling pathway. AKEX0011 ameliorated inflammation and fibrosis by inhibition of the activation of the p38 signaling pathway. **(C)** Macrophage polarization: In the pathophysiology of silicosis, the macrophage polarization disorder occupies an important position. AKEX0011 reduced macrophage polarization through suppressing NF-κB and PPAR-γ pathways.

In summary, AKEX0011 as a novel N-arylpyridone compound has demonstrated good therapeutic effects against silicosis both *in vivo* and *in vitro*, and the results suggest that its therapeutic effects are partially stronger than pirfenidone, a drug that has been approved for treatment of idiopathic pulmonary fibrosis. Our studies showed that AKEX0011 acts by mediating the ASK1-p38 pathway and macrophage polarization regulation (Figure 10). In addition to therapeutic efficacy, our results also suggest that the ASK1-p38 pathway is a potential therapeutic target for silicosis. However, the animal and cell models cannot fully mimic human silicosis. Future research is needed to explore if AKEX0011 would have any clinical application in the treatment of silicosis in human patients.

## Data Availability

The raw data supporting the conclusions of this article will be made available by the authors, without undue reservation.

## Supplementary Material

Supplementary Figure S1 Gate strategy of FACS.

Supplementary Figure S2 AKEX0011 administration dose-dependently reduced pulmonary inflammation and fribrosis a H&E staining of lung sections (original magnification 100x) b Masson's trichrome staining of lung sections (original magnification 100x). There were 7 experimental groups: PBS control (abbreviated as “Control” in the graphs), PBS+100 mpk AKEX0011 (abbreviated as “PBS+AKEX 100), silica+vehicle (abbreviated as “Silica”), silica+10 mpk AKEX0011 (abbreviated as “Si+AKEX 10”), silica+30 mpk AKEX0011 (abbreviated as “Si+AKEX 30”), silica+100 mpk AKEX0011 (abbreviated as “Si+AKEX 100”)in the following experiments of Supplementary Figures S1-S2.

Supplementary Figure S3 AKEX0011 administration dose-dependently reduced lung dysfunction a-d Lung function parameters improvement: IC, inspiratory capacity; Rrs, respiratory resistance; Ers, elastic resistance; G, tissue damping.

Supplementary Table S1 ELISA kits information.

Supplementary Table S2 ELISA kits information.

Supplementary Table S3 Primers for Quantitative Real-Time PCR.
