# Corrigendum: A novel N-arylpyridone compound alleviates the inflammatory and fibrotic reaction of silicosis by inhibiting the ASK1-p38 pathway and regulating macrophage polarization

**DOI:** 10.3389/fphar.2022.1109002

**Published:** 2023-01-18

**Authors:** Mingming Fan, Huijuan Xiao, Dingyun Song, Lili Zhu, Jie Zhang, Xinran Zhang, Jing Wang, Huaping Dai, Chen Wang

**Affiliations:** ^1^ Department of Respiratory Medicine, The Second Hospital of Jilin University, Jilin, China; ^2^ Department of Pulmonary and Critical Care Medicine Center of Respiratory Medicine, China-Japan Friendship Hospital, Capital Medical University, National Center for Respiratory Medicine, National Clinical Research Center for Respiratory Diseases, Institute of Respiratory Medicine, Chinese Academy of Medical Sciences, Peking Union Medical College, Beijing, China; ^3^ Department of Pulmonary and Critical Care Medicine, China-Japan Friendship School of Clinical Medicine, Peking University, Beijing, China; ^4^ Institute of Clinical Medical Sciences, China-Japan Friendship Hospital, Beijing, China; ^5^ State Key Laboratory of Medical Molecular Biology, Institute of Basic Medical Sciences Chinese Academy of Medical Sciences, School of Basic Medicine Peking Union Medical College, Beijing, China

**Keywords:** silicosis, pulmonary fibrosis, AKEX0011, macrophage polarization, pirfenidone

In the original article, there was a mistake in the legend for Figure 9 as published. The labelling of Figure 9 with macrophages is misleading as we used a RAW264.7 macrophage cell line. The correct legend appears below:

## FIGURE 9

AKEX0011 inhibited RAW264.7 from secreting pro-inflammatory cytokines, blocked p38 MAPK signaling, and reduced silica-induced apoptosis and M1 polarization. There were eight cell groups: PBS Control (abbreviated as “Control” in the graphs), PBS + AKEX0011 (200 μg/ml) (abbreviated as “AKEX”), Silica pre, Silica pre + AKEX0011 (100 μg/ml) (abbreviated as “Si pre + AKEX L”), and Silica pre + AKEX0011 (200 μg/ml) (abbreviated as “Si pre + AKEX H”), Silica post, Silica post + AKEX0011 (100 μg/ml) (abbreviated as “Si post + AKEX L”), and Silica post + AKEX0011 (200 μg/ml) (abbreviated as “Si post + AKEX H”). **(A−D)** IL-6 IL-1β, TNF-α, and TGF-β in cell supernatant detected by ELISA (n = 3). **(E)** Apoptosis (Annexin V+/PI− and Annexin V+/PI+) detection by FACS in each experimental group. **(F–H)** WB and quantification of P-p38, p38, P-ASK1, and ASK1. β-actin was used as a loading control. **(I–J)** M1 (F4/80 + CD86+) and M2 (F4/80 + CD163+) macrophage proportions detected by FACS in RAW264.7 and statistical analysis (n = 3). **(K)** WB of iNOS P-p65 p65. **(L**, **N)** Quantification of band densities fromWB images in (k), (n = 3). **(M)** mRNA levels of iNOS in lung tissues detected by qPCR (n = 3). All data were presented as mean ± SEM; *p < .05, **p < .01, ***p < .001, and ****p < .0001.

The authors apologize for this error and state that this does not change the scientific conclusions of the article in any way. The original article has been updated.

