# Addendum: A novel N-Arylpyridone compound alleviates the inflammatory and fibrotic reaction of silicosis by inhibiting the ASK1-p38 pathway and regulating macrophage polarization

**DOI:** 10.3389/fphar.2022.1108989

**Published:** 2023-01-18

**Authors:** Mingming Fan, Huijuan Xiao, Dingyun Song, Lili Zhu, Jie Zhang, Xinran Zhang, Jing Wang, Huaping Dai, Chen Wang

**Affiliations:** ^1^ Department of Respiratory Medicine, The Second Hospital of Jilin University, Jilin, China; ^2^ National Center for Respiratory Medicine, National Clinical Research Center for Respiratory Diseases, Department of Pulmonary and Critical Care Medicine Center of Respiratory Medicine, Chinese Academy of Medical Sciences, Peking Union Medical College, China-Japan Friendship Hospital, Capital Medical University, Institute of Respiratory Medicine, Beijing, China; ^3^ Department of Pulmonary and Critical Care Medicine, China-Japan Friendship School of Clinical Medicine, Peking University, Beijing, China; ^4^ Institute of Clinical Medical Sciences, China-Japan Friendship Hospital, Beijing, China; ^5^ State Key Laboratory of Medical Molecular Biology, School of Basic Medicine Peking Union Medical College, Institute of Basic Medical Sciences Chinese Academy of Medical Sciences, Beijing, China

**Keywords:** AKEX0011, macrophage polarization, pirfenidone, pulmonary fibrosis, silicosis

## Missing information

Add information about chemical formula, Table 2, polarization and antibody list.

**TABLE 2:** (Antibody list) Primary antibodies for Western Blot.

Antibodies	Cat No.	Manufacturer	Sources of species
Fibronectin	ab2413	abcam	Rabbit
Collagen-Ⅰ	66761-1-Ig	proteintech	Mouse
IκBα	#4814	cell signaling technology	Mouse
p38	#8690	cell signaling technology	Rabbit
P-p38	#4511	cell signaling technology	Rabbit
Arginase-1	ab233548	abcam	Rabbit
ASK-1	67072-1-Ig	proteintech	Rabbit
P-ASK-1	28846-1-AP	proteintech	Rabbit
NF-kB	ab32536	abcam	Rabbit
P-NF-kB	ab76302	abcam	Rabbit
iNOS	18985-1-AP	proteintech	Rabbit
β-Actin	ab8226	abcam	Mouse

## Chemical formula

Chemical structure of AKEX0011

Molecular formula C18H14N5O2F3, mass 389.3 g/mol, CAS:1590403-33-0.
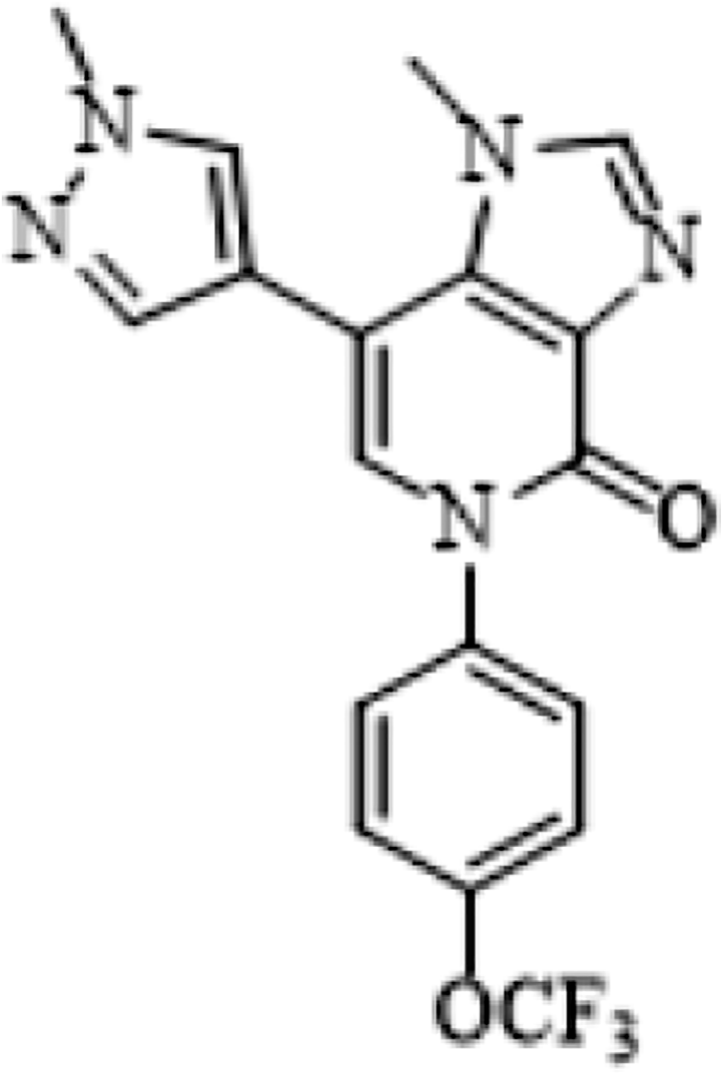


## Polarization

RAW 264.7 cells are a macrophage-like, Abelson leukemia virus-transformed cell line derived from BALB/c mice. This macrophage cell line is commonly used to study phagocytosis, apoptosis, inflammation, as well as M1/M2 polarization. Polarization of M1/M2 phenotype can be determined by the expression of specific M1 (CD80, CD86) and M2 (CD206, CD163) markers detected by flow cytometry. In the *in vitro* experiments of our study, we detected the expression of CD86 and CD163 in RAW264.7 cells of each group by flow cytometry to explore macrophage polarization. Our flow cytometry results confirmed that silica induced polarization of RAW264.7 from M0 macrophages toward certain M1 subtype (F4/80 + CD86^+^), but have no effect on the polarization towards M2 subtype (F4/80 + CD163+).

