# Supplementary figures and images for: A Novel N-Arylpyridone Compound Alleviates the Inflammatory and Fibrotic Reaction of Silicosis by Inhibiting the ASK1-p38 Pathway and Regulating Macrophage Polarization
Source: Front Pharmacol. 2022 Mar 23;13:848435. doi: 10.3389/fphar.2022.848435 (PMC8983992; doi:10.3389/fphar.2022.848435)

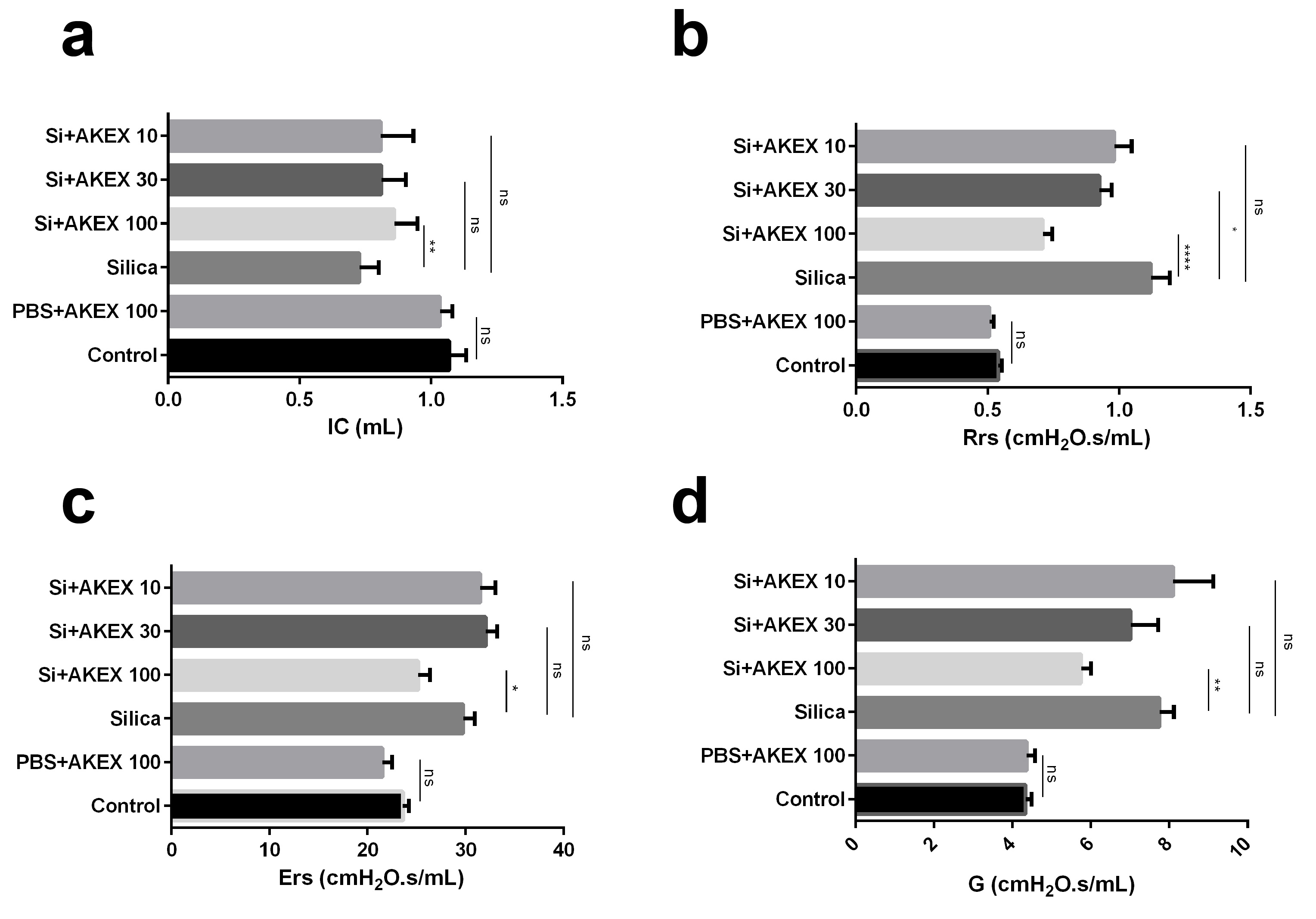

Supplement: Supplementary file 1 [file Image3.jpg]

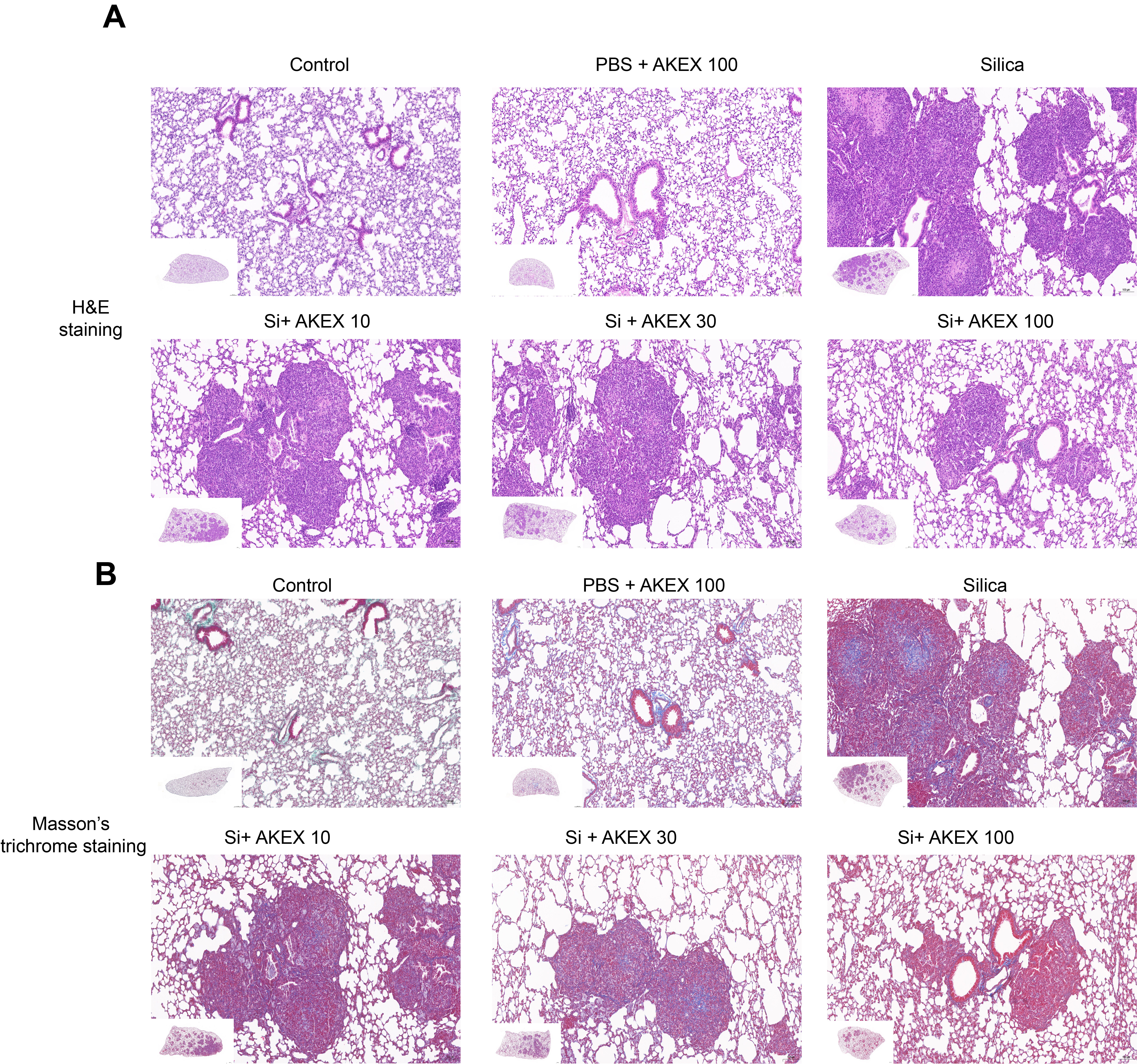

Supplement: Supplementary file 2 [file Image2.jpg]

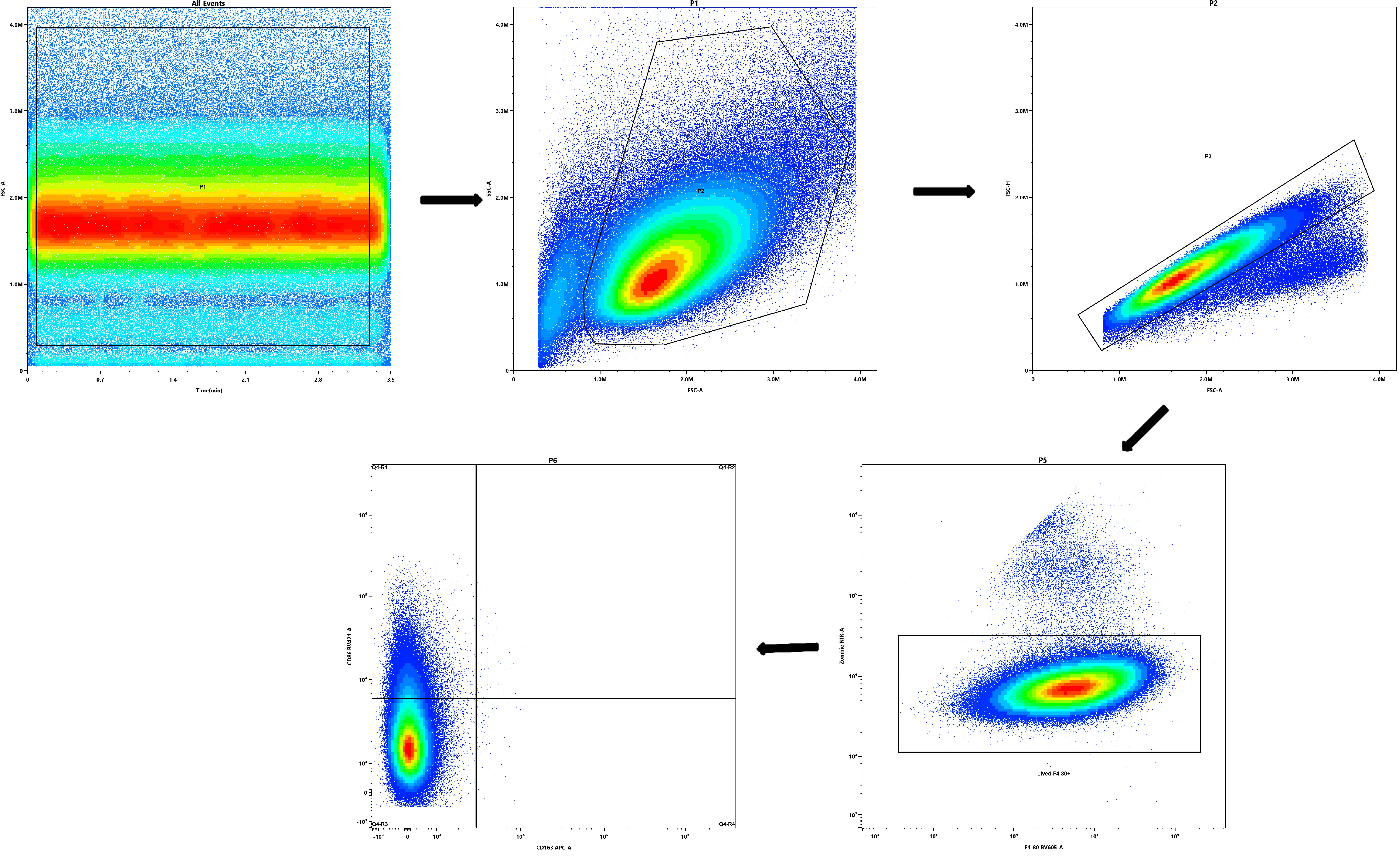

Supplement: Supplementary file 6 [file Image1.jpg]
