# Supplementary material for: A Novel N-Arylpyridone Compound Alleviates the Inflammatory and Fibrotic Reaction of Silicosis by Inhibiting the ASK1-p38 Pathway and Regulating Macrophage Polarization
Source: Front Pharmacol. 2022 Mar 23;13:848435. doi: 10.3389/fphar.2022.848435 (PMC8983992; doi:10.3389/fphar.2022.848435)
Supplement: Supplementary file 4 [file Table2.docx]

| **Supplementary material Table 2** | | |  |
| --- | --- | --- | --- |
| ELISA kits information. |  |  |  |
| Parameters | Cat No. | Manufacturer | species |
| IL-1β | MLB00C | R&D Systems | Mouse |
| IL-6 | M6000B | R&D Systems | Mouse |
| Hydroxyproline | E03H0014 | BLUE GENE | Mouse |
| TNF-α | MTA00B | R&D Systems | Mouse |
| TGF-β | E03T0009 | BLUE GENE | Mouse |
| IL-4 | E03I0007 | BLUE GENE | Mouse |
| IL-10 | E03I0023 | BLUE GENE | Mouse |
