# Supplementary material for: A Novel N-Arylpyridone Compound Alleviates the Inflammatory and Fibrotic Reaction of Silicosis by Inhibiting the ASK1-p38 Pathway and Regulating Macrophage Polarization
Source: Front Pharmacol. 2022 Mar 23;13:848435. doi: 10.3389/fphar.2022.848435 (PMC8983992; doi:10.3389/fphar.2022.848435)
Supplement: Supplementary file 5 [file Table3.docx]

| **Supplementary material Table 3** | |  |
| --- | --- | --- |
| Primers for Quantitative Real-Time PCR. | |  |
| Gene |  | Sequence 5’----3’(Mouse) |
| β-Actin | Foward | CATTGCTGACAGGATGCAGAAGG |
|  | Reverse | TGCTGGAAGGTGGACAGTGAGG |
| IL-1β | Foward | ACCTAGCTGTCAACGTGTGG |
|  | Reverse | TCAAAGCAATGTGCTGGTGC |
| IL-6 | Foward | CTCATTCTGCTCTGGAGCCC |
|  | Reverse | TTGTGAAGTAGGGAAGGCCG |
| Collagen-Ⅰ | Foward | GAGCAGACGGGAGTTTCTCCT |
|  | Reverse | CTTCTTGGCCATGCGTCAG |
| Fibronectin | Foward | CGGGAAGGTACTGTCCCATA |
|  | Reverse | GGAAAAGTCCTGAGGTGGGG |
| TNF-α | Foward | CTACTCCTCAGAGCCCCCAG |
|  | Reverse | TGACCACTCTCCCTTTGCAG |
| iNOS | Foward | CACCAAGCTGAACTTGAGCG |
|  | Reverse | CGTGGCTTTGGGCTCCTC |
| Arg1 | Foward | CCAGAAGAATGGAAGAGTCAGTGT |
|  | Reverse | GCAGATATGCAGGGAGTCACC |
| α-SMA | Foward | GTCCCAGACATCAGGGAGTAA |
|  | Reverse | TCGGATACTTCAGCGTCAGGA |
